# Processing technology optimization for tofu curded by fermented yellow whey using response surface methodology

**DOI:** 10.1002/fsn3.2331

**Published:** 2021-05-18

**Authors:** Zhanrui Huang, Wanying He, Liangzhong Zhao, Haiyu Liu, Xiaojie Zhou

**Affiliations:** ^1^ College of Food and Chemical Engineering Hunan Provincial Key Laboratory of Soybean Products Processing and Safety Control Shaoyang University Shaoyang China

**Keywords:** boiling‐to‐filtering method, fermented yellow whey, process optimization, soybean product, tofu

## Abstract

The technological applications utilized for tofu processing are diverse and complex, resulting in different yields and quality characteristics of tofu. The current study investigated the gel‐forming principle of soybean protein coagulated using fermented yellow whey (FYW) to produce tofu. The effects of several processing parameters (soybean‐to‐water ratio, boiling temperature, boiling time, and FYW content) on the yield and protein content of tofu produced by the boiling‐to‐filtering method (BFM) were studied and optimized using response surface methodology. Results indicated significant differences in yield and protein content of tofu using different processing parameters, with FYW content being the most significant (*p* < .05). Optimum processing parameters of the BFM were found to be: soybean‐to‐water ratio of 1:5 (kg:kg), boiling time 6.1 min, boiling temperature 105°C, and FYW content of 26%. Under optimum conditions, tofu's yield and protein content were 235.17 g/100 g and 10.60%, respectively, and these were 47.93 g/100 g and 4.16% higher than those before optimization. This study provides practical technical support and a theoretical basis for the standardized industrial production of high‐yield and high‐protein tofu.

## Graphical abstract

Response surface methodology was designed to determine optimal process parameters for producing tofu by boiling‐to‐filtering method. Under optimum parameters, the yield and protein content of tofu were 235.17 g/100 g and 10.60%, respectively, and these were 47.93 g/100 g and 4.16% higher than those before optimization. This study provides an effective technical support and theoretical basis for the standardized industrial production of high‐yield and high‐protein tofu.

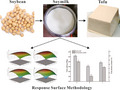

## 1 INTRODUCTION

Tofu, also called soybean curd, is a highly gelatinous product derived from soybean protein rich in protein, isoflavones, vitamins, minerals, and other nutrients. The high nutritional value makes tofu potentially one of the most nutritious foods available for commercial development and consumption (Li et al., 2012; Sun et al., 2019). The high‐protein value of tofu makes it an indispensable element in human diets. Long‐term consumption of tofu can reduce the occurrence of some common chronic diseases such as hypertension, hyperlipidemia, coronary heart disease, and cardio‐cerebrovascular sclerosis (Friedman & Brandon 2001; Huang et al., 2019).

Tofu processing has a long history, including diverse and complex technological applications. A typical process includes soybean soaking (6 hr), homogenization (30 min), boiling (95–115°C and 3–12 min), separating, solidification (40 min), and pressing. The treatment of soybean homogenate (soymilk) is critical in tofu production (Rosenthal et al., 2003; Wang, Meng, et al., 2020). The various processing methods play a decisive role in the extraction of soymilk's nutrients and stability, significantly impacting the quality and flavor of tofu produced (Rekha & Vijayalakshmi, 2013; Wang, Meng, et al., 2020). Consideration of boiling and filtering results in two technological approaches for making tofu: the filtering‐to‐boiling method (FBM) and boiling‐to‐filtering method (BFM) (Yu et al., 2015). FBM is most commonly used to produce tofu on Chinese farms and companies due to the simple equipment requirements and easy operation (Zhang et al., 2017). However, soymilk and tofu produced by FBM have poor taste and stability, and the resulting protein content is lower than that obtained from other processes (Yu et al., 2015). BFM improves the retention rate of protein and fat in soybean, and quality characteristics (such as color, taste, and texture) of soymilk and tofu are a higher standard than using FBM (Elias et al., 1986; Yu et al., 2015). BFM is currently widely used in developed countries such as Japan, Denmark, and Sweden.

Several studies have shown that the yield and quality of tofu are closely related to factors such as the thickness of the soybean homogenate, order of boiling and separation, heating temperature and time, the quantity of coagulant and coagulated time (Shi et al., 2015; Tsai et al., 1981). Therefore, understanding the correlation between different processing technological applications and the tofu quality evaluation index, and determining optimal processing parameters of tofu, are essential for assisting tofu producers in improving the yield and texture of tofu while realizing an automatic production of tofu.

Soybean yellow whey (SYW) is the yellow liquid produced by soybean protein's reaction with coagulant or is produced during tofu pressing (Wu et al., 2021). Statistics indicate that every 1 tonne of soybean processed by a factory produces 2–5 tonne SYW, which may pollute the environment and waste resources (Chua & Liu, 2019). Therefore, the efficient utilization of SYW has attracted a great deal of attention in the food industry. Previously, SYW fermented by multiple strains prepared a fermented yellow whey (FYW) that was rich in organic acids (lactic acid, acetic acid, citric acid, etc.) and probiotics (lactic acid bacteria, acetic acid bacteria, etc.) (Liu et al., 2020). When the concentration of soybean protein is lower than 10%, coagulants (such as calcium sulfate and magnesium chloride) must be added to promote soybean protein binding, transforming soymilk coagulate into tofu (Guo et al., 2002; Chen et al., 2016). FYW has recently been commonly used as a tofu coagulant. The solidification mechanism involves modifying soymilk pH to the electric point of soybean protein to form a gel, producing tofu (Guo et al., 2002). Studies have shown that FYW can improve the extraction rate of protein and polysaccharides from tofu. Tofu products made by FYW have high nutritional value, sound firmness, increased water retention, and a unique flavor (Prabhakaran et al., 2005; Wu et al., 2021).

The tofu processing using BFM is optimized in the current study by developing and optimizing the gel‐forming principle of soybean protein coagulation by FYW. Outcomes of variation of the soybean‐to‐water ratio, boiling temperature and time, and FYW content on the yield and protein content of tofu were investigated using single‐factor experiments. The response surface design optimization experiment allowed the determination of optimal process parameters so that the yield and protein content of tofu were improved. These results also provide informative and productive technical support and a theoretical basis for the standardized industrial production of tofu using FYW.

## 2 MATERIALS AND METHODS

### 2.1 Materials and chemicals

Canadian non‐GMO soybean (protein content 38%) was purchased from the Wanyue Import and Export Trade Co. Ltd (Yueyang, China). FYW (total acid content: 4.49 ± 0.1 g/L, protease content: 9.51 ± 0.2 U/ml) was obtained from the Hunan Provincial Key Laboratory of Soybean Products Processing and Safety (Shaoyang, China). All other chemical reagents were purchased from the Nanjing Chemical Reagent Co. Ltd.

### 2.2 Selection of variables in BFM

The experiment was designed using the Box‐Behnken principle with the single factors of soybean‐to‐water ratio, boiling temperature and time, and FYW content assessed independently. The regression equation and data model were established by response surface methodology (RSM), and the tofu yield and protein content were used as indices to optimize the BFM process parameters. Five soybean‐to‐water ratios were selected: 1:3, 1:4, 1:5, 1:6, and 1:7 (kg:kg); boiling temperatures of 95°C, 100°C, 105°C, 110°C, and 115°C were assessed; boiling times were set at 0 min, 3 min, 6 min, 9 min, and 12 min; and FYM contents of 15%, 20%, 25%, 30%, and 35% were considered. While other factors remain constant, each factor's effects on the yield and protein content of tofu were explored.

### 2.3 Preparation of fermented yellow whey tofu by BFM

**FIGURE 1:**
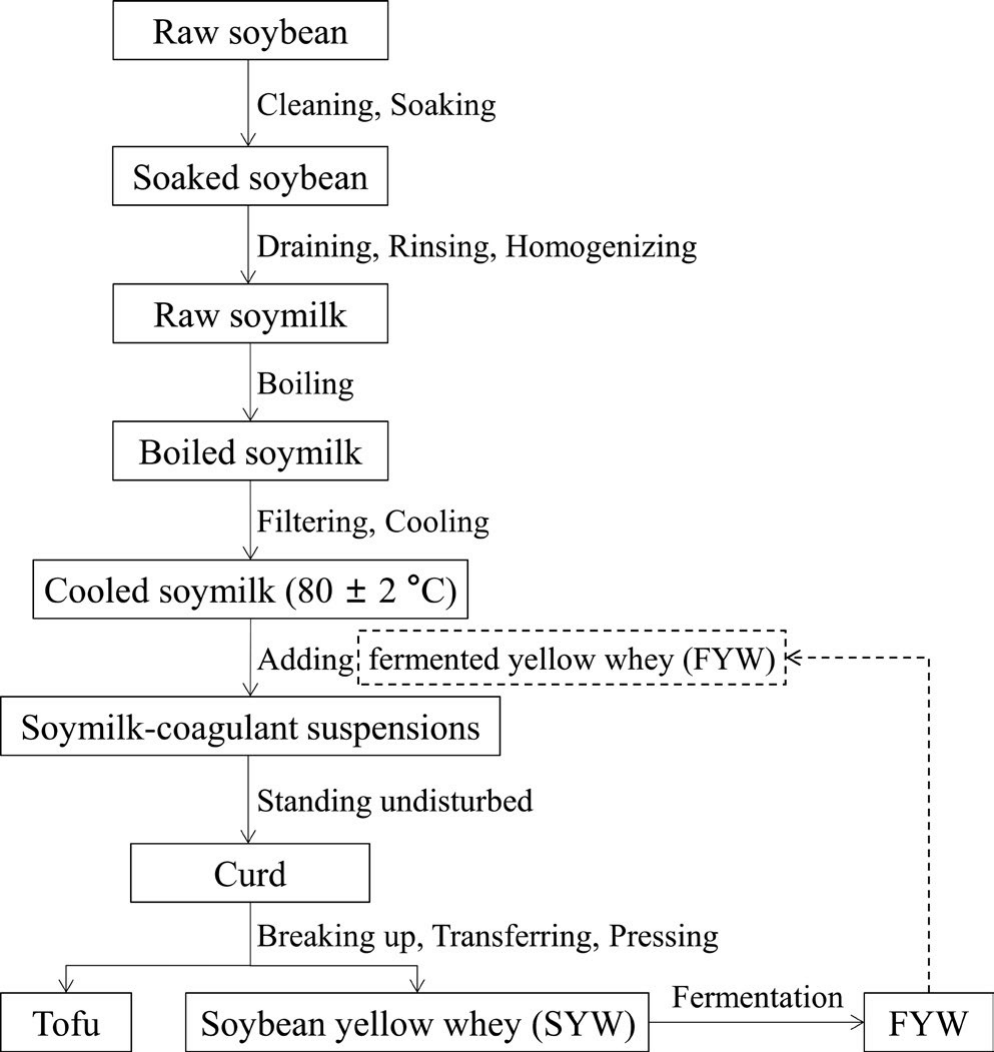
Schematic flow chart of the production of tofu from soybeans

FYW tofu was produced according to Qiao et al., (2010), as shown in Figure 1. Soybeans (250 g) were washed, soaked overnight in water at room temperature, drained, rinsed, and homogenized with water in a soymilk grinder (SJJ‐20, Kangdeli Machinery Equipment Manufacturing Co. Ltd, Beijing, China). Raw soymilk was heated, filtered, and cooled (80 ± 2°C) in a 0.2 t soymilk‐integrated equipment (MZJJ‐1, Kangdeli Machinery Equipment Manufacturing Co., Ltd). The FYW solution was slowly poured into the integrated equipment while stirring slowly, and FYW was no longer added once coagulation of the soymilk occurred. Soymilk‐coagulant suspensions were allowed to stand for 20 min (80 ± 2°C) to ensure that coagulation had been completed. Curds were broken and gently transferred to a perforated container (11.1 × 7.1 × 5.5 cm) lined with a single layer of cheesecloth. The SYW was drained off for 10 min, and the curd was pressed for 8 min using an automatic press machine (ZZY‐800, Kangdeli Machinery Equipment Manufacturing Co. Ltd). After pressing, tofu and SYW were weighed separately and stored for further analysis.

### 2.4 Determining tofu yield

Tofu yield was determined according to Cai and Chang (1999). Each group of three 5.0 g tofu samples was placed into a constant weight bottle, weighed, and recorded (EL204, Mettler Toledo International Trade (Shanghai) Co., Ltd, China). Soybean samples were dried at 101–105°C for four h (GZX‐9140MBE, Shanghai Boxun Industry & Commerce Co., Ltd, China), removed, cooled for 30 min, weighed, and recorded. Tofu yield was calculated using the following equation:

$$Y=\frac{m1}{m0}\times 100.$$

where *Y* represents tofu yield (g / 100g), *m*_1_ tofu quality (g), and *m*_0_ soybean quality (g).

### 2.5 Determining protein content in tofu

The protein content of tofu was determined using the Kjeldahl method, according to Huang et al., (2020). The protein conversion factor was 6.25. Protein content was calculated using the following equation:

$$X=\frac{(V_{1}‐V_{2})\times c\times 0.0140}{\frac{m}{100}\times 10}\times F\times 100\text{\textbackslash{}\%}\;$$

where *X* represents tofu protein content (%), *V*_1_ the volume of hydrochloric acid standard solution consumed in sample titration (ml), *V*_2_ the volume of hydrochloric acid standard solution consumed in blank titration (ml), *c* the concentration of hydrochloric acid standard titration solution (mol / L), 0.0140 the mass of nitrogen in 1.0 ml hydrochloric acid (0.1 mol/L) standard titration solution, *m* the quality of the sample (g), and *F* the conversion factor of nitrogen to protein.

### 2.6 Experimental design of response surface methodology

The most influential factors of the BFM process were assessed using a single‐factor test in the surface response experiment. The yield and protein content of tofu were used as reference indices to optimize the investigation, and the process was repeated three times for each group.

### 2.7 Validation test

Three experimental groups were set up to produce tofu according to the RSM optimization scheme and process parameters: two BFM groups (before and after process optimization) and an FBM group. Each group data point was repeated three times. The differences in tofu yield and protein content were evaluated between the three experimental groups.

### 2.8 Statistical analysis

Data are presented as the mean ±standard deviation (*SD*). Statistical analysis and analysis of variance (ANOVA) of the data were done using SPSS 22.0, origin 9.1, and design expert 8.0 software. Significant and highly significant outcomes were defined as *p* < .05 and *p* < .01, respectively, to identify significant differences between the groups.

## 3 RESULTS AND DISCUSSION

### 3.1 Effects of soybean‐to‐water ratio on yield and protein content of tofu

**FIGURE 2:**
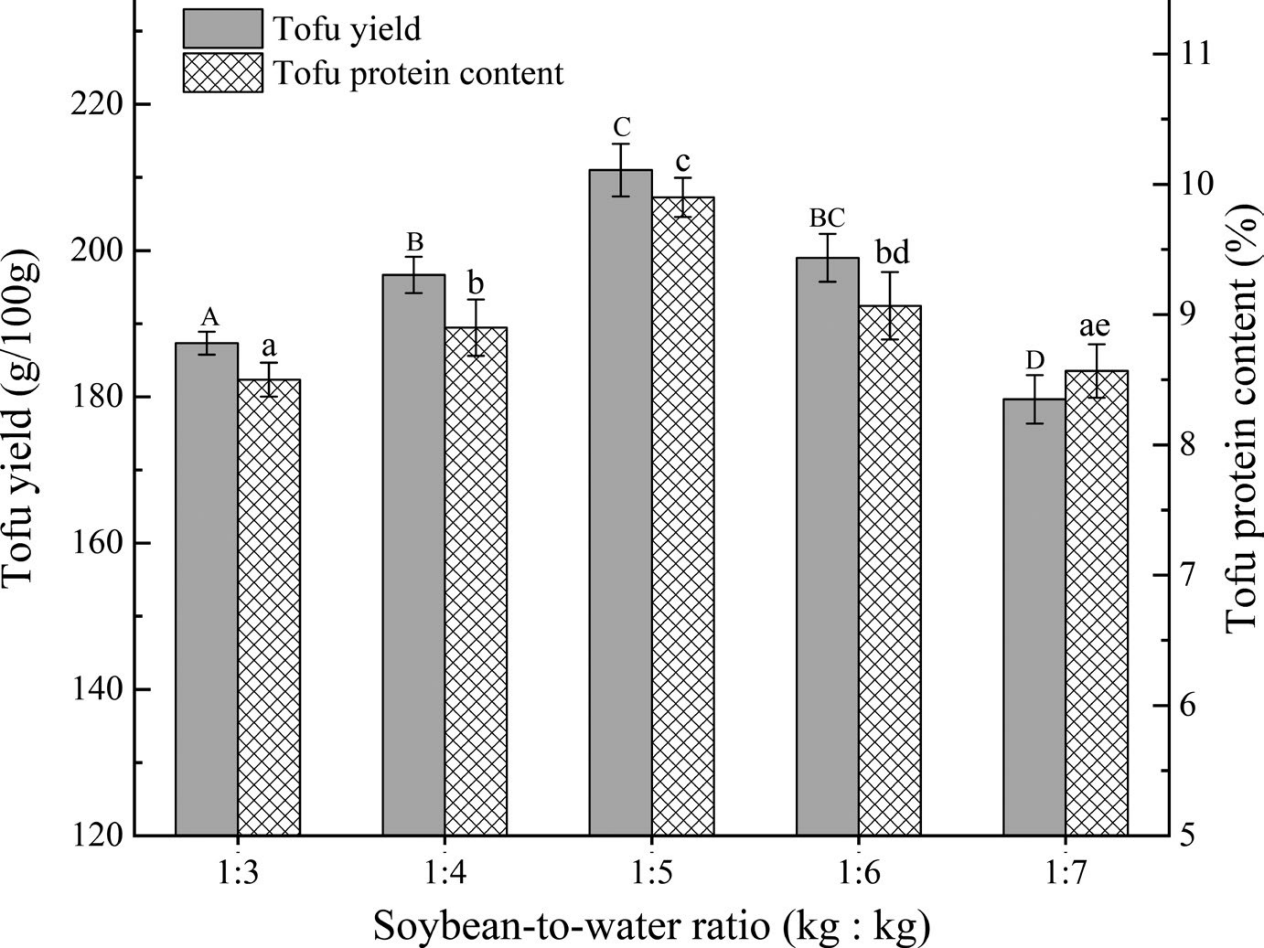
Effects of different soybean‐to‐water ratios on tofu yield and protein content in FBBT. The lowercase letters above the columns represent statistically significant differences between treatments (*p* < .05)

The effects of different soybean‐to‐water ratios on yield and protein content in BFM are shown in Figure 2. The yield and protein content within the tofu initially increased and subsequently decreased with decreasing soybean‐to‐water ratio. Statistical analysis showed a significant correlation between the soybean‐to‐water ratio and the tofu's quality, reaching the highest value when the ratio was 1:5 (kg:kg). This could be due to the soymilk protein concentration being largest when the ratio of soybean‐to‐water was high, which is conducive to a more complete reaction of protein with H^+^ and protease in FYW, promoting the maximal formation of protein gel that in turn enhanced the yield of tofu (Hu et al., 2013; Nik et al., 2011). As the ratio of soybean‐to‐water (water content increased) decreased, the protein content in soymilk became diluted, reducing H^+^ and proteinase in FYW, which leads to an inadequate formation of protein gel. The inadequate protein gel adversely impacted the 3D network structure of tofu (Wang, Yang, et al., 2020), resulting in lower tofu yield and protein content. Subsequently, the soybean‐to‐water ratios of 1:4, 1:5, and 1:6 (kg:kg) were selected for further optimization according to the design principle of RSM (select three better parameters).

### 3.2 Effects of boiling temperature on yield and protein content of tofu

**FIGURE 3:**
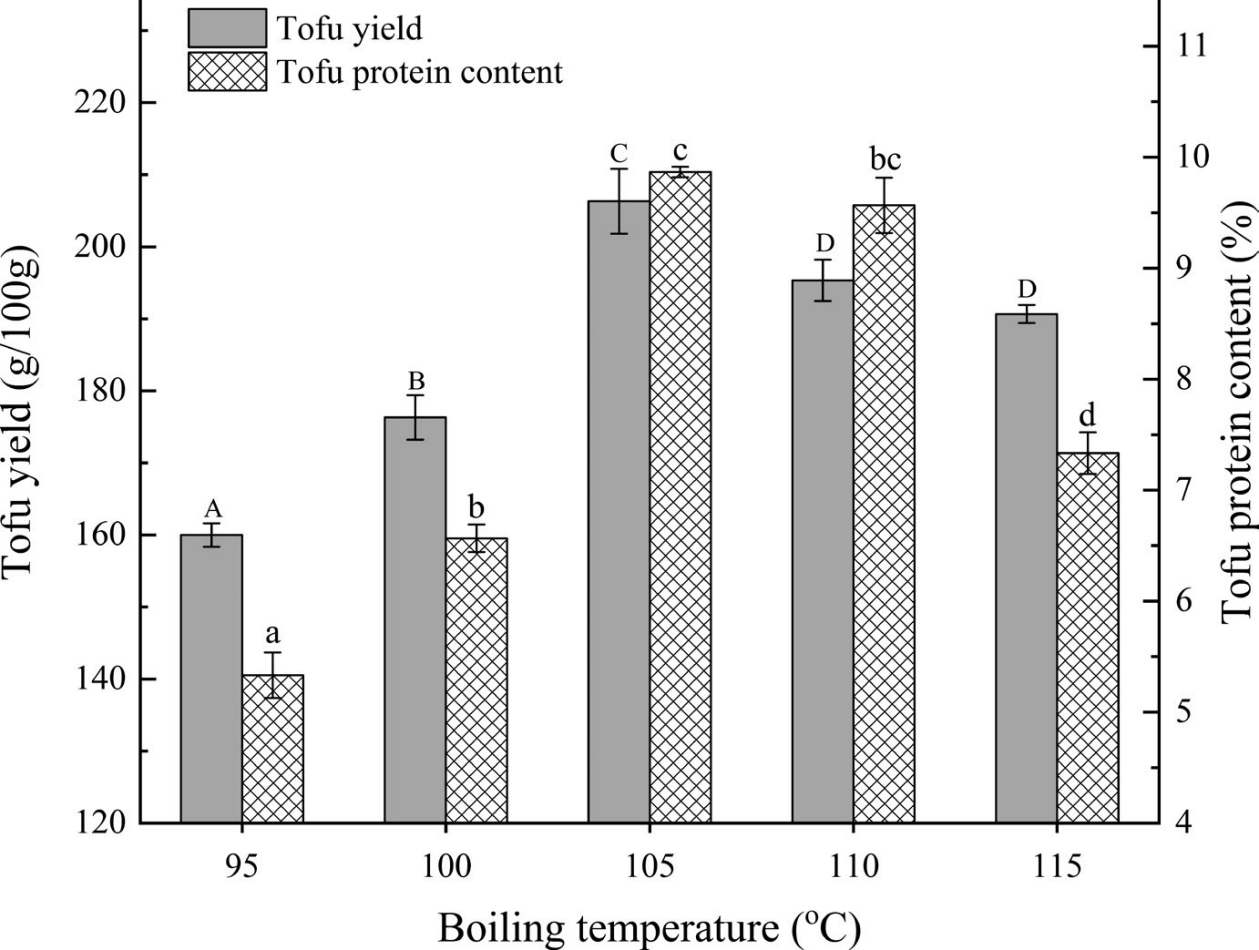
Effects of different boiling temperatures on tofu yield and protein content in BFM. The lowercase letters above the columns represent statistically significant differences between treatments (*p* < .05)

The yield and protein content of tofu in BFM were measured as a function of different boiling temperatures, and the data are shown in Figure 3. Yield and protein content increased significantly with increasing boiling temperature from 95 to 105°C (*p* < .05), reaching the maxima at 105°C, 206.33 g/100 g, and 9.87%, respectively. Speculatively, as the boiling temperature increases, the molecular conformation of soybean protein changes from the natural β‐folded state to the expanded state (Du et al., 2018). The hydrophilic groups of soybean protein become fully exposed, which is conducive to increased solubility of soybean protein in water (Hui et al., 2000) and the diffusion of protein particles (Toda et al., 2007; Zhao et al., 2016), thus improving the yield and protein content. However, yield and protein content decreased slightly at 110–115°C. Potentially, the higher temperature causes denaturation of soymilk protein molecules (Shimoyamada et al., 2008), destruction of the proteins' secondary structure (Yang et al., 2010), and the inactivation of hydrophobic and active groups (Saio et al., 1971). Overall, the resulting gradual decline in the 3D network structure stability of tofu with increasing temperature (Wang & Damodaran, 1991) decreases yield and protein content. Therefore, boiling temperatures of 100°C, 105°C, and 110°C were selected for further optimization according to the design principle of RSM.

### 3.3 Effects of boiling time on yield and protein content of tofu

**FIGURE 4:**
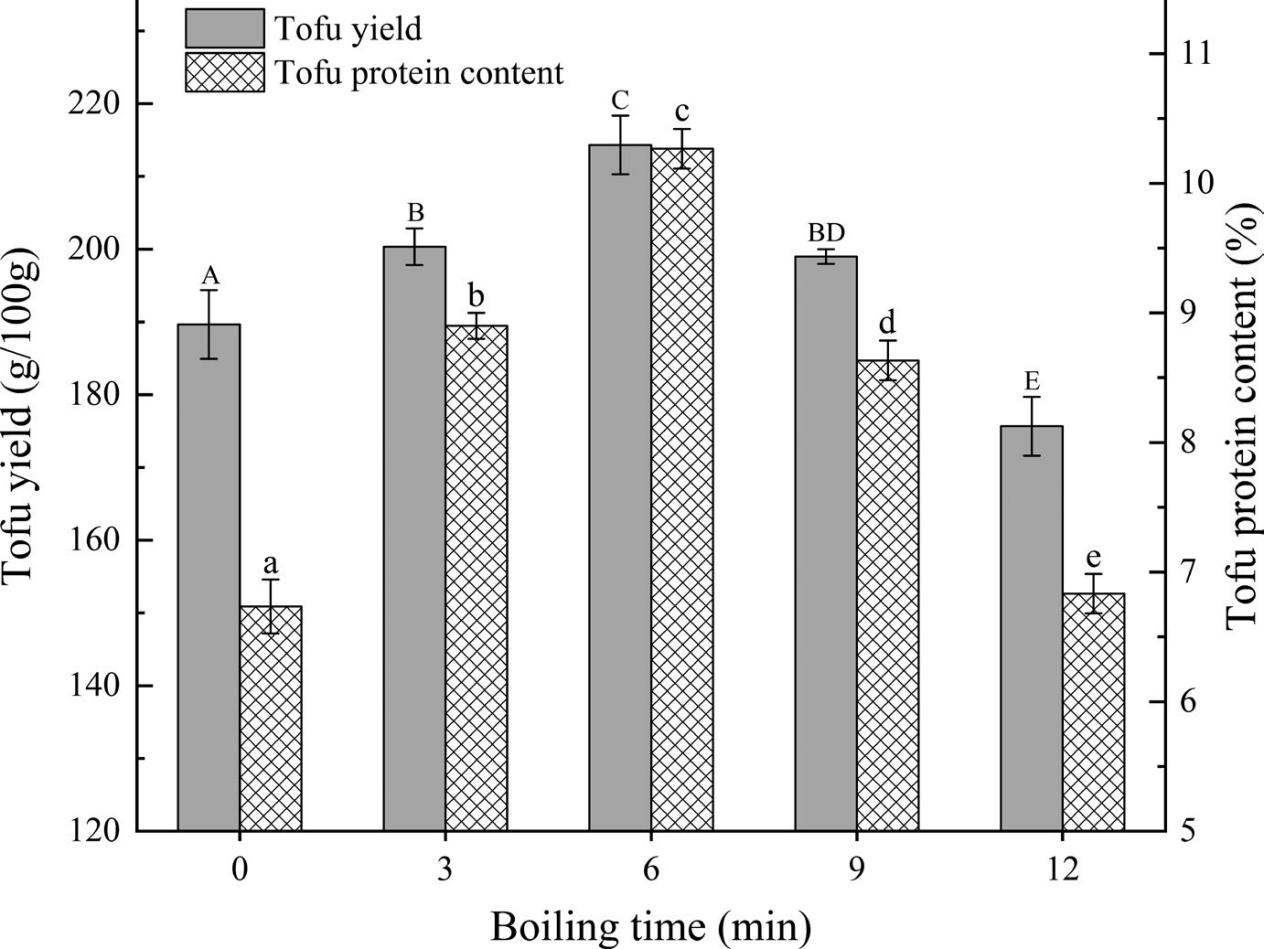
Effects of different boiling times on tofu yield and protein content in BFM. The lowercase letters above the columns represent statistically significant differences between treatments (*p* < .05)

The effects of different boiling times on the yield and protein content of tofu in BFM are shown in Figure 4. As boiling time was extended, yield increased slowly from 0 to 6 min but decreased significantly after 6 min (*p* < .05). The tofu's protein content showed a normal distribution trend and reached the maximum value of 10.27% at 6 min. When boiling time is ≤6 min, the sulfhydryl and hydrophobic groups within the protein molecules are exposed, enabling them to form aggregates through two sulfur bonds and hydrophobic interactions that can enhance the strength of protein gel and increase yield and protein content (Hsia et al., 2016; Wu et al., 2021). However, when the boiling time is extended past 6 min, excessive oxidation of sulfhydryl in soymilk protein molecules changed the 3D network structure of tofu (Renkema et al., 2002), resulting in a decreased yield. In contrast, the reaction between 7S and 11S subunits caused insoluble multi‐stage polymer and precipitation formation (Hsia et al., 2016; Hsiao et al., 2015), resulting in a decreased protein content. Therefore, boiling times of 3 min, 6 min, and 9 min were selected for further optimization according to the design principle of RSM.

### 3.4 Effects of FYW content on yield and protein content of tofu

**FIGURE 5:**
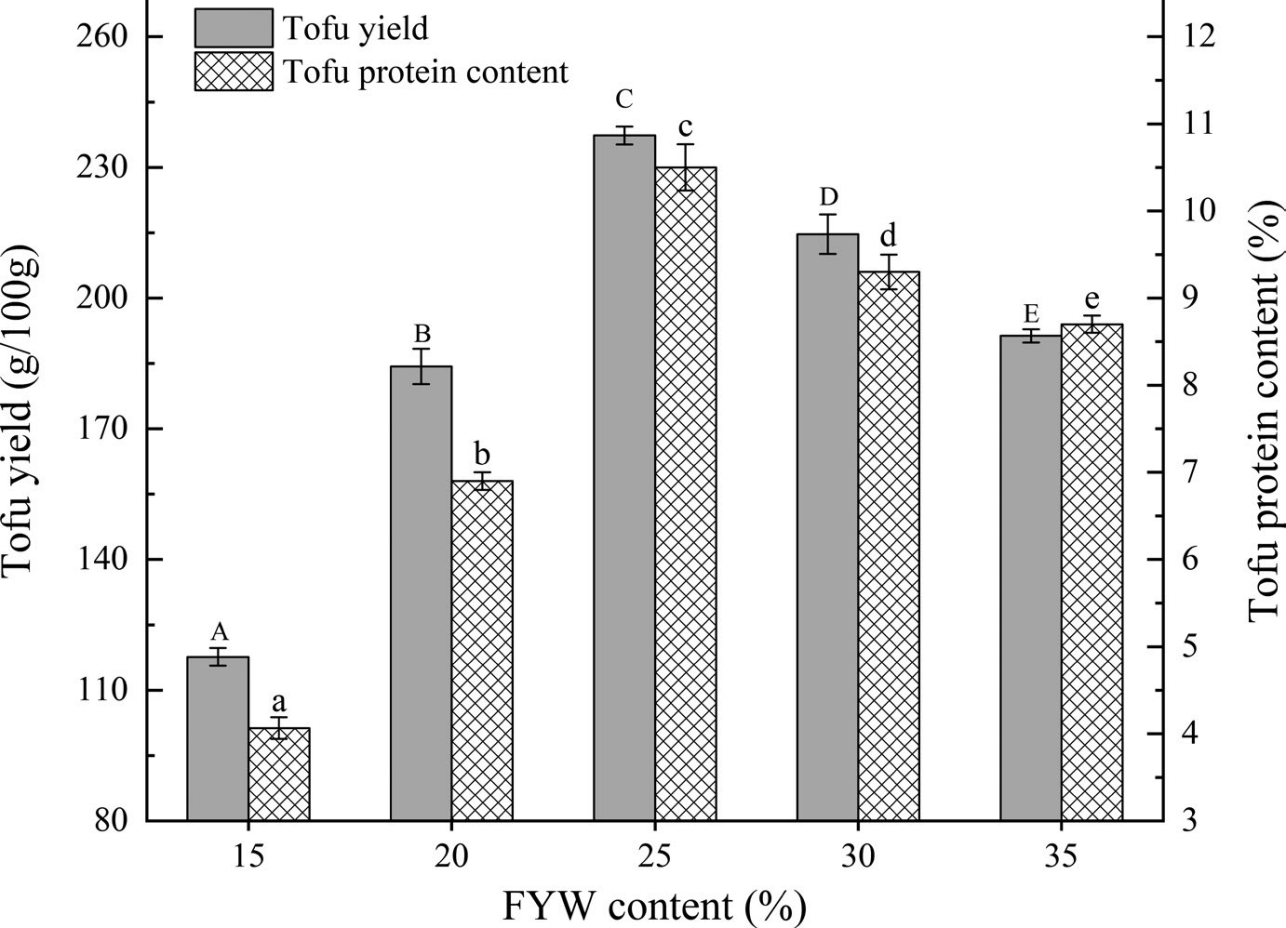
Effects of different FYW contents on tofu yield and protein content in BFM. The lowercase letters above the columns represent statistically significant differences between treatments (*p* < .05)

The results for varying the FYW contents on yield and protein content of tofu in BFM are shown in Figure 5. Yield and protein content increased significantly with increasing FYW content at 15%–25% (*p* < .05). FYW contains large quantities of organic acids such as lactic acid, which provides H^+^ to reduce soybean milk pH, in an environment where weakly acidic negative protein ions can easily obtain H^+^. Such symbiosis reduces the charge on the protein's surface resulting in electrical neutrality, thereby forming a more stable protein gel system (Grygorczyk & Corredig, 2013; Wu et al., 2021). However, yield and protein content decreased slightly when the FYW content was 30%–35%, possibly due to the increased H^+^ concentration interfering with coagulation forces between the soymilk protein molecules (Li et al., 2017; Ringgenberg et al., 2013), resulting in a loose 3D network gel structure of tofu and decreased yield and protein content. Therefore, FYW contents of 20%, 25%, and 30% were selected for further optimization according to the design principle of RSM.

### 3.5 Response surface optimization methodology results

RSM was applied to variations of the soybean‐to‐water ratio (A), boiling temperature (B), boiling time (C), and FYW content (D). Tofu yield and protein content were used as reference indices to optimize the experimental factors. Factor level settings and results of the RSM are shown in Table 1 and Table 2, respectively. Multiple regression analysis was applied to the data using the Design Expert 8.0 software. The quadratic polynomial regression equation of tofu yield (*Y*_1_) and protein content (*Y*_2_) was obtained as follows:

**TABLE 1:** Level of response surface methodology factors

Level	A—soybean‐to‐water ratio (kg : kg)	B—boiling time (min)	C—boiling temperature (^o^C)	D—FYW content (%)
−1	1:4	3	100	20
0	1:5	6	105	25
1	1:6	9	110	30

**TABLE 2:** Response surface methodology results of process optimization

Test number	Soybean‐to‐water ratio	Boiling time	Boiling temperature	FYW content	Tofu yield (g / 100g)	Tofu protein content (%)
1	−1	−1	0	0	190.47 ± 1.14	8.97 ± 0.51
2	−1	0	−1	0	186.62 ± 1.25	8.21 ± 0.29
3	−1	0	0	−1	180.14 ± 0.37	6.61 ± 0.11
4	−1	0	0	1	195.04 ± 2.63	9.63 ± 0.42
5	−1	0	1	0	186.50 ± 1.71	8.59 ± 0.03
6	−1	1	0	0	194.39 ± 2.74	8.82 ± 0.44
7	0	0	1	−1	175.72 ± 1.15	6.24 ± 0.56
8	0	0	0	0	233.89 ± 0.31	10.49 ± 0.03
9	0	1	−1	0	183.90 ± 0.19	7.76 ± 0.43
10	0	1	0	−1	180.36 ± 4.72	6.84 ± 0.14
11	0	−1	0	1	192.64 ± 1.67	8.73 ± 0.03
12	0	−1	0	−1	169.61 ± 2.61	6.18 ± 0.32
13	0	0	0	0	228.52 ± 3.62	10.38 ± 0.15
14	0	0	1	1	186.24 ± 4.51	8.55 ± 0.12
15	0	1	0	1	190.27 ± 2.17	8.89 ± 0.15
16	0	0	−1	1	186.35 ± 4.47	7.71 ± 0.14
17	0	−1	1	0	187.33 ± 0.66	7.94 ± 0.28
18	0	0	0	0	232.28 ± 3.38	10.38 ± 0.11
19	0	0	0	0	229.42 ± 1.18	10.41 ± 0.08
20	0	1	1	0	185.80 ± 4.58	8.54 ± 0.56
21	0	0	−1	−1	160.35 ± 1.29	5.82 ± 0.16
22	0	−1	−1	0	174.49 ± 1.06	7.31 ± 0.54
23	0	0	0	0	236.24 ± 2.07	10.51 ± 0.24
24	1	0	0	−1	179.30 ± 1.02	7.43 ± 0.44
25	1	0	0	1	200.11 ± 2.69	8.48 ± 0.55
26	1	1	0	0	196.22 ± 2.02	8.94 ± 0.07
27	1	−1	0	0	197.87 ± 0.21	8.13 ± 0.35
28	1	0	−1	0	180.24 ± 1.62	7.67 ± 0.34
29	1	0	1	0	195.45 ± 2.57	8.72 ± 0.52

*Y*_1_ = 231.60 – 1.33A + 1.58B + 3.75C + 8.83D + 1.25AB ‐ 3.75AC – 1.50AD – 2.75BC – 3.25BD – 3.75CD – 16.26A^2^ – 21.38B^2^ – 28.13C^2^ – 27.01D^2^.

*Y*_2_ = 10.43 + 0.12A + 0.21B + 0.34C + 1.07D – 0.24AB – 0.17AC + 0.49AD + 0.037BC – 0.12BD + 0.11CD – 0.64A^2^ – 1.03B^2^ – 1.53C^2^ – 1.77D^2^.

The ANOVA analysis of RSM for tofu yield and protein content showed that "*p* values" for the tofu yield and protein content models were less than 0.01, indicating that the regression models were significant (*p* < .01) (Table 3). The values of "Lack of Fit" were 0.9943 and 0.1554, inferring that "Lack of Fit" was not significant relative to the pure error (*p* > .05); therefore, the models were reliable. The *R* ^2^ of the tofu yield and protein content models were 0.9949 and 0.9974, respectively. The Adj.*R* ^2^ was 0.9899 and 0.9948, respectively, confirming that the two models reflect every single factor's relationship, with a high fitting degree and small experimental error. Such data align with the change of 98.57% tofu yield and 98.92% tofu protein content response values, respectively, which can be used to analyze and predict the BFM process.

**TABLE 3:** Variance analysis of the response surface methodology for tofu yield and protein content

Source of variance	Tofu yield				Tofu protein content			
	Mean square	*F* value	*p* value	Significant	Mean square	*F* value	*p* value	Significant
Model	784.64	196.92	<.0001	^**^	3.51	384.63	<.0001	^**^
A—soybean‐to‐water ratio	21.33	5.35	.0364	*	0.18	19.46	.0006	^**^
B—boiling time	30.08	7.55	.0157	*	0.53	58.44	<.0001	^**^
C—boiling temperature	168.75	42.35	<.0001	^**^	1.40	153.46	<.0001	^**^
D—FYW content	936.33	234.99	<.0001	^**^	13.80	1512.13	<.0001	^**^
AB	6.25	1.57	.2309		0.23	25.24	.0002	^**^
AC	56.25	14.12	.0021	^**^	0.11	12.29	.0035	^**^
AD	9.00	2.26	.1551		0.97	106.29	<.0001	^**^
BC	30.25	7.59	.0155	*	5.63 × 10^–3^	0.62	.4455	
BD	42.25	10.60	.0057	^**^	0.063	6.85	.0203	*
CD	56.25	14.12	.0021	^**^	0.044	4.83	.0453	*
A^2^	1714.60	430.31	<.0001	^**^	2.64	289.47	<.0001	^**^
B^2^	2,965.93	744.36	<.0001	^**^	6.91	756.80	<.0001	^**^
C^2^	5,133.95	1,288.47	<.0001	^**^	15.20	1665.07	<.0001	^**^
D^2^	4,731.57	1,187.49	<.0001	^**^	20.42	2,237.57	<.0001	^**^
Lack of fit	0.9943 (Not significant)				0.1554 (Not significant)			
*R* ^2^	.9949				.9974			
Adj. *R* ^2^	.9899				.9948			

* indicates significant differences (*p <* .05); ^**^indicates extremely significant differences (*p <* .01).

The derived regression equation and ANOVA analysis of the two models showed that the correlation order of each factor on yield was as follows: D (FYW content) > C (boiling temperature) > B (boiling time) > A (soybean‐to‐water ratio). The order of influence on protein content was: D (FYW content) > C (boiling temperature) > B (boiling time) > A (soybean‐to‐water ratio). Overall, the results showed that FYW content had the greatest effect on yield and protein content, followed by boiling temperature, with the soybean‐to‐water ratio having the least effect. FYW content exerted a major outcome on the Y value, indicating that the FYW content determined whether tofu gel was formed or not. When too low in content, the FYW could not react with soymilk sufficiently to promote gelatin formation. When too high, FYW would lead to the production of a large quantity of SYW and nutrient loss, resulting in a decrease in tofu yield and protein content (Wu et al., 2021). Secondly, the boiling temperature played a secondary role in the Y value, presumably due to the close relationship between boiling temperature and protein denaturation (Shimoyamada et al., 2008; Shin et al., 2015).

### 3.6 Analysis of interaction between factors

**FIGURE 6:**
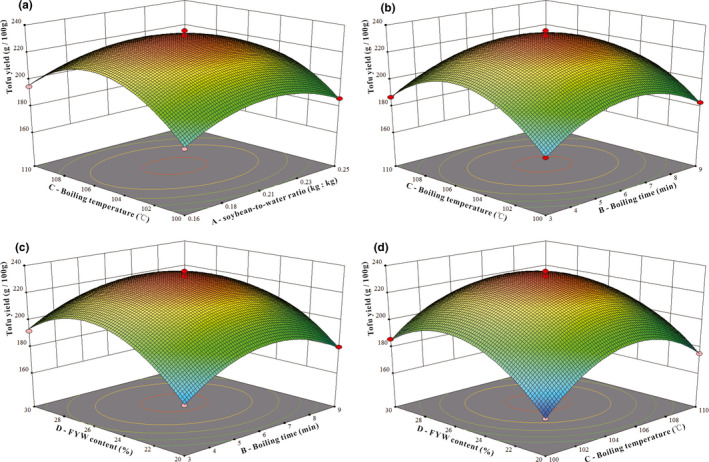
Effects of the interaction of two factors on tofu yield. (a) represents C—boiling temperature and A—soybean‐to‐water ratio. (b) represents C—boiling temperature and B—boiling time. (c) represents D—FYW content and B—boiling time. (d) represents D—FYW content and C—boiling temperature

**FIGURE 7:**
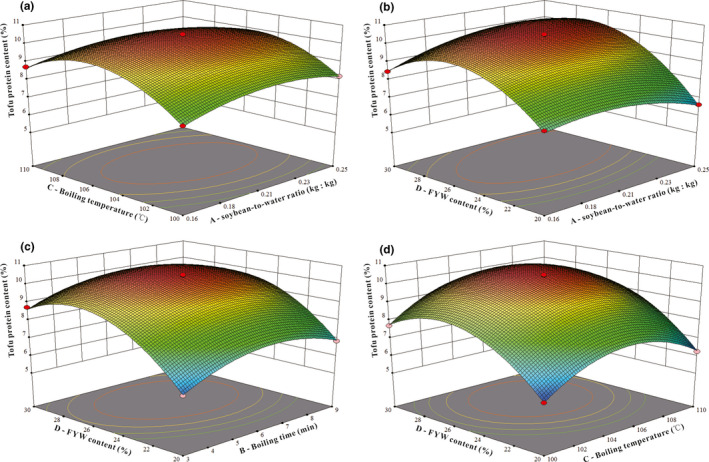
Effects of the interaction of two factors on tofu protein content. (a) represents C—boiling temperature and A—soybean‐to‐water ratio. (b) represents D—FYW content and A—soybean‐to‐water ratio. (c) represents D—FYW content and B—boiling time. (d) represents D—FWY content and C—boiling temperature

The RSM model can directly and accurately describe the interaction between two variables (factors), expressing the influence of different variables (factors) on a particular index (Ferrari et al., 2013). The interactive effects of the soybean‐to‐water ratio, boiling time and temperature, and FYW content on the yield and protein content of tofu are shown in Figures 6 and 7. Every two factors affected each other in a parabolic relationship, and there was a unique, optimal RSM value. Additionally, the steeper the 3D curve, the more significant the effect of this factor on yield or protein content (Yu et al., 2016). In Figure 6, the correlations between A (soybean‐to‐water ratio) and C (boiling temperature), B (boiling time) and D (FYW content), and C (boiling temperature) and D (FYW content) had highly significant effects on yield (*p* < .01). The interaction between B (boiling time) and C (boiling temperature) has a significant impact on yield (*p* < .05), which is consistent with the significance test results of partial regression coefficients in *Y*_1_. In Figure 7, the interactions between A (soybean‐to‐water ratio) and B (boiling time), A (soybean‐to‐water ratio) and C (boiling temperature), A (soybean‐to‐water ratio) and D (FYW content) had highly significant effects on protein content (*p* < .01). Correlations between B (boiling time) and D (FYW content), and C (boiling temperature) and D (FYW content) had significant effects on the concentration of tofu protein (*p* < .05), which is again consistent with the significance test results of partial regression coefficients in *Y*_2_.

### 3.7 Effects of different technological applications on yield and protein content of tofu

From the optimization scheme obtained by Design Expert 8.0 software, optimal process parameters of tofu yield are as follows: soybean‐to‐water ratio is 1:5 (kg:kg), boiling time is 6.06 min, boiling temperature of 105.29°C, and FYW content of 25.80%. Optimal process parameters of tofu protein content are as follows: soybean‐to‐water ratio of 1:4.8 (kg: kg), boiling time is 6.14 min, boiling temperature of 105.40°C, and FYW content was 26.18%. The R^2^ of the two derived equations for Y1 and Y2 was compared, resulting in process parameters: a ratio of soybean‐to‐water is 1:5 (kg:kg), boiling time is 6.1 min, the boiling temperature of 105°C, and FYW content of 26%.

**FIGURE 8:**
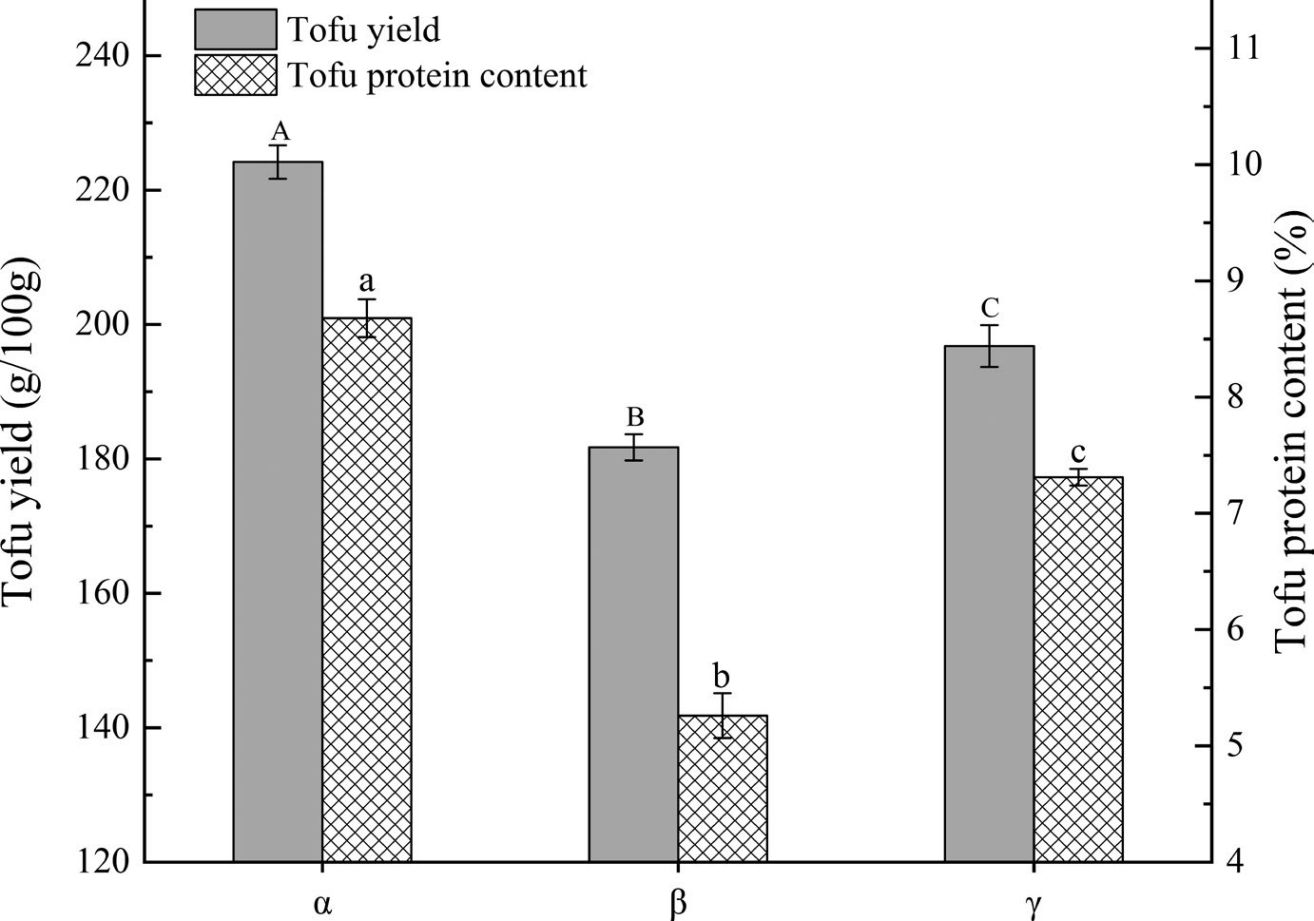
Effects of different processing technological applications on yield and protein content of tofu: (α) represents optimized BFM; (β) represents BFM before optimization; and (γ) represents FBM. The different lower case letters above the columns represent statistically significant differences between treatments (*p* < .05)

Three experimental groups where (α) used the optimal process parameters obtained above, β (BFM before optimization) and γ (FBM), were followed through the tofu producing process (Figure 8). Yield and protein content in α were 235.17 g/100 g and 10.60%, respectively, 47.63 g/100 g and 4.16% higher than those of β. These outcomes confirmed that the optimized process conditions validate that they produced higher yields and protein contents. Additionally, the yield and protein content in α were increased by 26.39 g/100g and 2.38%, respectively, compared with γ, confirming that the BFM method is more productive than FBBT. It is hypothesized that the expansion of cellulose in the soybean homogenate during the heating process is conducive to improved filtration efficiency, leading to retaining protein more effectively (Guo & Ono, 2005). Equally, the protein, unsaturated fats, and reducing sugar in the soybean homogenate were fully dissolved, conducive to the formation of protein gel and tofu yield (Yu et al., 2015).

## 4 CONCLUSIONS

The current study investigates the effects of different parameters of BFM on the yield and protein content of tofu using single‐factor experimentation based on the gel‐forming principle of soybean protein coagulated by FYW. Response surface methodology was designed to determine optimal process parameters for producing tofu using the BFM approach. Results showed that the yield and protein content were significantly affected by different process parameters, and variations in FYW content are the most significant. Optimum processing parameters of BFM are as follows: the ratio of soybean‐to‐water of 1:5 (kg:kg), boiling time is 6.1 min, the boiling temperature of 105°C, and content of FYW is 26%. Yield and protein content produced under the optimal process parameters were 235.17 g/100g and 10.60%, respectively, with both outcomes being an improvement upon the original BFM process and FBBT. This study provides productive and practical technical support, together with a sound theoretical basis, for standardized industrial production of high‐protein and high‐yield tofu.

## CONFLICT OF INTEREST

The authors declare that they have no conflict of interest in this study.

## AUTHOR CONTRIBUTION

**Zhanrui Huang:** Conceptualization (lead); Methodology (lead); Validation (lead); Writing‐original draft (lead); Writing‐review & editing (lead). **Wanying He:** Formal analysis (supporting); Writing‐original draft (supporting). **Liangzhong Zhao:** Conceptualization (supporting); Writing‐review & editing (supporting). **Haiyu Liu:** Formal analysis (supporting); Methodology (supporting); Software (supporting). **Xiaojie Zhou:** Conceptualization (supporting); Validation (supporting).

## References

[fsn32331-bib-0001] Cai, T. D. , & Chang, K. C. (1999). Processing effect on soybean storage proteins and their relationship with tofu quality. Journal of Agricultural and Food Chemistry, 47, 720–727. 10.1021/jf980571z 10563959

[fsn32331-bib-0002] Chen, Y. C. , Chen, C. C. , Chen, S. T. , & Hsieh, J. F. (2016). Proteomic profiling of the coagulation of milk proteins induced by glucono‐delta‐lactone. Food Hydrocolloids, 52, 137–143. 10.1016/j.foodhyd.2015.06.005

[fsn32331-bib-0003] Chua, J. Y. , & Liu, S. Q. (2019). Soy whey: More than just wastewater from tofu and soy protein isolate industry. Trends in Food Science & Technology, 91, 24–32. 10.1016/j.tifs.2019.06.016

[fsn32331-bib-0004] Du, Y. L. , Huang, G. Q. , Wang, H. O. , & Xiao, J. X. (2018). Effect of high coacervation temperature on the physicochemical properties of resultant microcapsules through induction of Maillard reaction between soybean protein isolate and chitosan. Journal of Food Engineering, 234, 91–97. 10.1016/j.jfoodeng.2018.04.020

[fsn32331-bib-0005] Elias, E. E. , Malcolm, C. B. , & Lamartine, F. H. (1986). Effect of boiling treatment of soymilk on the composition, yield, texture and sensory properties of tofu. Canadian Institute of Food Science and Technology Journal, 19, 53–56. 10.1016/S0315-5463(86)71416-8

[fsn32331-bib-0006] Ferrari, I. , Alamprese, C. , Mariotti, M. , Lucisano, M. , & Rossi, M. (2013). Optimisation of cake fat quantity and composition using response surface methodology. International Journal of Food Science & Technology, 48, 468–476. 10.1111/ijfs.12018

[fsn32331-bib-0042] Friedman, M. , & Brandon, D. L. (2001). Nutritional and health benefits of soy proteins. Journal of Agricultural and Food Chemistry, 49, 1069–1086. 11312815 10.1021/jf0009246

[fsn32331-bib-0007] Grygorczyk, A. , & Corredig, M. (2013). Acid induced gelation of soymilk, comparison between gels prepared with lactic acid bacteria and glucono‐delta‐lactone. Food Chemistry, 141, 1716–1721. 23870883 10.1016/j.foodchem.2013.03.096

[fsn32331-bib-0008] Guo, S. T. , & Ono, T. (2005). The role of composition and content of protein particles in soymilk on tofu curding by glucono‐δ‐lactone or calcium sulfate. Journal of Food Science, 70, 258–262. 10.1111/j.1365-2621.2005.tb07170.x

[fsn32331-bib-0009] Guo, S. T. , Tsukamoto, C. , Takahasi, K. , Yagasaki, K. , Nan, Q. X. , & Ono, T. (2002). Incorporation of soymilk lipid into soy protein coagulum by the addition of calcium chloride. Journal of Food Science, 67, 3215–3219. 10.1111/j.1365-2621.2002.tb09568.x

[fsn32331-bib-0010] Hsia, S. Y. , Hsiao, Y. H. , Li, W. T. , & Hsieh, J. F. (2016). Aggregation of soy protein‐isoflavone complexes and gel formation induced by glucono‐delta‐lactone in soymilk. Scientific Reports, 6, 3571801–3571811. 10.1038/srep35718 PMC5071761 27760990

[fsn32331-bib-0011] Hsiao, Y. H. , Yu, C. J. , Li, W. T. , & Hsieh, J. F. (2015). Coagulation of beta‐conglycinin, glycinin and isoflavones induced by calcium chloride in soymilk. Scientific Reports, 5, 13018. 26260443 10.1038/srep13018 PMC4542527

[fsn32331-bib-0012] Hu, H. , Fan, X. , Zhou, Z. , Xu, X. , Fan, G. , Wang, L. , Huang, X. , Pan, S. , & Zhu, L. (2013). Acid‐induced gelation behavior of soybean protein isolate with high intensity ultrasonic pre‐treatments. Ultrason Sonochemistry, 20, 187–195. 10.1016/j.ultsonch.2012.07.011 22925550

[fsn32331-bib-0013] Huang, J. , Yokoyama, W. H. , & Kim, Y. (2019). Soy noodles processed from soy flour or tofu affects antioxidant content, lipid accumulation in 3T3‐L1 cells, and plasma lipids in hamsters. Journal of Food Processing and Preservation, 43, 1–8. 10.1111/jfpp.13871

[fsn32331-bib-0014] Huang, Z. , Wang, Y. , Sun, L. , Wang, X. , Lu, P. , Liang, G. , Pang, H. , Wu, Q. , Gooneratne, R. , & Zhao, J. (2020). Effects of T‐2 toxin on the muscle proteins of shrimp (Litopenaeus vannamei) ‐ a proteomics study. Journal of the Science of Food and Agriculture, 100, 119–128. 31441054 10.1002/jsfa.10001

[fsn32331-bib-0015] Hui, L. K. , Easa, A. M. , & Ismail, N. (2000). Effects of thermal treatments on texture of soy protein isolate tofu. Journal of Food Processing and Preservation, 24, 275–286. 10.1111/j.1745-4549.2000.tb00419.x

[fsn32331-bib-0016] Li, C. , Rui, X. , Zhang, Y. , Cai, F. , Chen, X. , & Jiang, M. (2017). Production of tofu by lactic acid bacteria isolated from naturally fermented soy whey and evaluation of its quality. LWT‐Food Science and Technology, 82, 227–234. 10.1016/j.lwt.2017.04.054

[fsn32331-bib-0017] Li, J. , Qiao, Z. , Tatsumi, E. , Saito, M. , Cheng, Y. , & Yin, L. (2012). A novel approach to improving the quality of bittern‐solidified tofu by W/O controlled‐release coagulant. 2: Using the improved coagulant in tofu processing and product evaluation. Food and Bioprocess Technology, 6, 1801–1808. 10.1007/s11947-012-0849-y

[fsn32331-bib-0018] Liu, H. Y. , Fan, L. , Zhao, L. Z. , Deng, Y. X. , Xie, C. P. , Shen, G. X. , Ou, H. Y. , Lin, Z. Q. , Yu, K. , & Mo, X. (2020). Process optimization of producing tofu based on the technology of pulped by soymilk and dreg repeated curing of soybean whey fermented liquid. Science and Technology of Food Industry, 41, 189‐195, 209. (in China)

[fsn32331-bib-0019] Nik, A. M. , Alexander, M. , Poysa, V. , Woodrow, L. , & Corredig, M. (2011). Effect of soy protein subunit composition on the rheological properties of soymilk during acidification. Food Biophysics, 6, 26–36. 10.1007/s11483-010-9172-1

[fsn32331-bib-0020] Prabhakaran, M. P. , Perera, C. O. , & Valiyaveettil, S. (2005). Quantification of isoflavones in soymilk and tofu from South East Asia. International Journal of Food Properties, 8, 113–123. 10.1081/JFP-200048055

[fsn32331-bib-0021] Qiao, Z. , Chen, X. D. , Cheng, Y. , Liu, H. , Liu, Y. , & Li, L. (2010). Microbiological and chemical changes during the production of acidic whey, a traditional chinese tofu‐coagulant. International Journal of Food Properties, 13, 90–104. 10.1080/10942910802180190

[fsn32331-bib-0022] Rekha, C. R. , & Vijayalakshmi, G. (2013). Influence of processing parameters on the quality of soycurd (tofu). Journal of Food Science and Technology, 50, 176–180. 10.1007/s13197-011-0245-z 24425905 PMC3550946

[fsn32331-bib-0023] Renkema, J. M. S. , Gruppen, H. , & Vliet, T. V. (2002). Influence of pH and ionic strength on heat‐induced formation and rheological properties of soy protein gels in relation to denaturation and their protein compositions. Journal of Agricultural and Food Chemistry, 50, 6064–6071. 10.1021/jf020061b 12358481

[fsn32331-bib-0024] Ringgenberg, E. , Alexander, M. , & Corredig, M. (2013). Effect of concentration and incubation temperature on the acid induced aggregation of soymilk. Food Hydrocolloids, 30, 463–469. 10.1016/j.foodhyd.2012.05.011

[fsn32331-bib-0025] Rosenthal, A. , Deliza, R. , Cabral, L. M. C. , Cabral, L. C. , Farias, C. A. A. , & Domingues, A. M. (2003). Effect of enzymatic treatment and filtration on sensory characteristics and physical stability of soymilk. Food Control, 14, 187–192. 10.1016/S0956-7135(02)00087-7

[fsn32331-bib-0026] Saio, K. , Kajikawa, M. , & Watanabe, T. (1971). Food processing characteristics of soybean proteins.2. Effect of sulfhydryl groups on physical properties of tofu‐gel. Agricultural and Biological Chemistry, 35, 890–898.

[fsn32331-bib-0027] Shi, X. , Li, J. , Wang, S. , Zhang, L. , Qiu, L. , Han, T. , Wang, Q. , Chang, S. K. , & Guo, S. (2015). Flavor characteristic analysis of soymilk prepared by different soybean cultivars and establishment of evaluation method of soybean cultivars suitable for soymilk processing. Food Chemistry, 185, 422–429. 10.1016/j.foodchem.2015.04.011 25952888

[fsn32331-bib-0028] Shimoyamada, M. , Tsushima, N. , Tsuzuki, K. , Asao, H. , & Yamauchi, R. (2008). Effect of heat treatment on dispersion stability of soymilk and heat denaturation of soymilk protein. Food Science and Technology Research, 14, 32–38. 10.3136/fstr.14.32

[fsn32331-bib-0029] Shin, W. K. , Yokoyama, W. H. , Kim, W. , Wicker, L. , & Kim, Y. (2015). Change in texture improvement of low‐fat tofu by means of low‐fat soymilk protein denaturation. Journal of the Science of Food and Agriculture, 95, 1000–1007. 10.1002/jsfa.6780 24924689

[fsn32331-bib-0030] Sun, D. , Li, T. , Ma, L. , Zhang, F. , Li, A. , & Jiang, Z. (2019). Effect of selective thermal denaturation and glycosylation on the textural properties and microstructure of vegetable tofu. Journal of Food Process Engineering, 42, 1–9. 10.1111/jfpe.13001

[fsn32331-bib-0031] Toda, K. , Chiba, K. , & Ono, T. (2007). Effect of components extracted from okara on the physicochemical properties of soymilk and tofu texture. Journal of Food Science, 72, C108–C113. 10.1111/j.1750-3841.2006.00248.x 17995824

[fsn32331-bib-0032] Tsai, S. J. , Lan, C. Y. , Kao, C. S. , & Chen, S. C. (1981). Studies on the yield and quality characteristics of tofu. Journal of Food Science, 46, 1734–1737. 10.1111/j.1365-2621.1981.tb04474.x

[fsn32331-bib-0033] Wang, C. H. , & Damodaran, S. (1991). Thermal gelation of globular proteins: Influence of protein conformation on gel strength. Journal of Agricultural and Food Chemistry, 39, 433–438. 10.1021/jf00003a001

[fsn32331-bib-0034] Wang, F. , Meng, J. , Sun, L. , Weng, Z. , Fang, Y. , Tang, X. , Zhao, T. , & Shen, X. (2020). Study on the tofu quality evaluation method and the establishment of a model for suitable soybean varieties for Chinese traditional tofu processing. LWT ‐ Food Science Technology, 117, 108441. 10.1016/j.lwt.2019.108441

[fsn32331-bib-0035] Wang, Y. , Yang, X. , & Li, L. (2020). A new style of fermented tofu by Lactobacillus casei combined with salt coagulant. 3 Biotech, 10(2). 10.1007/s13205-019-2040-x PMC6994563 32099732

[fsn32331-bib-0036] Wu, H. , Dong, J. J. , Dai, Y. Q. , Liu, X. L. , Zhou, J. Z. , & Xia, X. D. (2021). Effects of lactic acid bacteria fermented yellow whey on the protein coagulation and isoflavones distribution in soymilk. Food Chemistry, 334, 127484. 32711263 10.1016/j.foodchem.2020.127484

[fsn32331-bib-0037] Yang, F. , Weng, D. , Huang, X. J. , Yao, X. L. , & Pan, S. Y. (2010). Research on the interaction of lipid and protein in SPI gel. European Food Research and Technology, 230, 467–473. 10.1007/s00217-009-1176-z

[fsn32331-bib-0038] Yu, H. S. , Chen, J. Z. , Zhang, W. , Wang, Y. H. , Liu, J. M. , Pu, C. H. , & Hu, Y. H. (2015). Relationship between nutritional components and texture profile of tofu from two preparation methods. Food Science, 36, 49–54.(in China).

[fsn32331-bib-0039] Yu, M. , Liu, H. Z. , Yang, Y. , Shi, A. M. , Liu, L. , Hu, H. , Wang, Q. , Yu, H. W. , & Wang, X. H. (2016). Optimising germinated conditions to enhance yield of resveratrol content in peanut sprout using response surface methodology. International Journal of Food Science and Technology, 51, 1754–1761.

[fsn32331-bib-0040] Zhang, B. Y. , Yang, Y. L. , Zhang, J. , Tang, L. , & Jiang, H. T. (2017). Effect of different soybean pretreatment methods on the quality of soybean milk. Food and Fermentation Industries, 43, 134–140.(in China).

[fsn32331-bib-0041] Zhao, H. , Li, W. , Qin, F. , & Chen, J. (2016). Calcium sulphate‐induced soya bean protein tofu‐type gels: Influence of denaturation and particle size. International Journal of Food Science and Technology, 51, 731–741. 10.1111/ijfs.13010

